# Psychological autopsy of 101 suicide cases from northwest region of India

**DOI:** 10.4103/0019-5545.39757

**Published:** 2008

**Authors:** B. S. Chavan, Gurvinder Pal Singh, Jaspreet Kaur, Reshma Kochar

**Affiliations:** Department of Psychiatry, Govt. Medical College and Hospital, Chandigarh, India

**Keywords:** Psychiatric illness, suicide, treatment

## Abstract

**Background:**

The present study was conducted by the Department Of Psychiatry, Govt. Medical College and Hospital, Chandigarh, to investigate suicide cases during the year 2003.

**Aim:**

To assess the socio-demographic characteristics, psychosocial factors, and psychiatric and physical comorbidity associated with completed suicide.

**Materials and Methods:**

One hundred one suicide cases were assessed using semi-structured proforma for recording socio-demographic profile, psychosocial variables, and treatment details.

**Results:**

Majority (59.4%) of suicide victims were in the age group of 20 to 29 years. Males (57.4%) slightly outnumbered females (42.57%) in this study. As many as 57.4% of the subjects had migrated from other parts of India. Hanging was the most common method used by the suicide victims (72.2%). Psycho-social stressors were found in 61 (60.3%) suicide victims. Psychiatric illness was found in 34 cases (33.6%). However, out of them only 16 (48.5%) suicide victims sought treatment prior to the attempt. As many as 57.4% of the subjects had shown behavioral change before the suicidal attempt.

**Conclusions:**

Our study suggests that specific focus in suicide prevention strategies should be on migrant population.

## INTRODUCTION

In depth study of the history of suicide prior to the suicidal act is known as psychological autopsy.[1] This is the most informative means of studying the nature and causes of suicide.[2] This method is commonly used in various studies to assess and manage suicidal patients.[3–4] Findings from such studies offer clues for planning suicide prevention strategies. Suicide rate in India is approximately 11.4 per 1 lakh in males and 8.0 per 1 lakh in females.[5] India and China are responsible for 30% of all cases of suicide worldwide.[5]

In an Indian study, it was reported that predominant suicidal victims were unemployed males, middle-aged and high school-educated subjects; and they were mostly from a rural background.[6] Even people with low suicidal intention may end up in completed suicides as a result of using more lethal methods and inadequate treatment.[7] In India, it is the comparatively younger people who are suicide victims.[8–9] This phenomenon of successful completion of suicide is a dangerous trend in India. While the population increase in the last decade was 25%, the suicide rate increased by 60%.[10]

In our country, there is a paucity of studies regarding psychological autopsy of suicide victims. Various psycho-social characteristics of suicide victims in the northwest region of India, which had been witnessing a large number of suicides, are inadequately studied. In the year 2003, Chandigarh, which is a small city with a population of 10 lakhs with the highest per capita income, witnessed a large number of suicides. One hundred thirty cases of suicide were reported in the media, and many more must have gone unreported. Many suicide victims were young school-going children. These developments raised an alarm for Chandigarh administration, and it was decided to investigate the problem and initiate interventions at different levels. Department of Psychiatry, Government Medical College and Hospital, Chandigarh, was made the nodal agency for assessment, training, and treatment. Hence in the present study, an attempt was made to investigate suicide cases that occurred in the year 2003, with the following aims and objectives:

1. To study the socio-demographic characteristics, psycho-social factors, and psychiatric and physical comorbidity associated with completed suicide.
2. To evaluate the method used in suicide completion.
3. To study the details of treatment sought prior to suicide.

## MATERIALS AND METHODS

### Sample

This study pertains to the 130 suicide cases that occurred in the general population residing in the Union Territory, Chandigarh, in the year 2003.

### Method of collection of data

There were three sources of information:

1. **Daily newspaper reporting:** All the successful suicides in the city which were reported in the leading newspapers were recorded. Help was taken from the newspaper section of the Central Library of Govt. Medical College, Chandigarh. In the library, a Library Restorer was requested to screen all the newspapers and record the cases of suicide and report it to the investigation team on a daily basis. The Central Library receives 12 newspapers published in English, Hindi, and Punjabi. Chandigarh being a small city with very active media, chances of suicide not being reported were very low as compared to the rest of the country.
2. **Local police department:** The local police department was requested to provide detailed records of all the suicide cases registered with them during the year 2003. Senior Superintendent of Police, Union Territory, Chandigarh, directed all the station house officers to provide all the details of suicide victims in their jurisdiction to the Department of Psychiatry.
3. **Office of the Registrar of Births and Deaths:** Information regarding reported deaths due to suicide and confirmation of addresses of suicide victims was also obtained from the Office of the Registrar of Births and Deaths, Union Territory, Chandigarh.

The information collected from these three sources was cross-checked to avoid any duplication.

### Tools

The following tools were used:

1. **Semi-structured performa to record socio-demographic profile:** This performa was used to collect the socio-demographic details of each suicide case (age, sex, marital status, educational qualifications, income, school and job history, family history, etc.)
2. **Semi-structured performa for psycho-social variables:** This performa assessed various psycho-social variables associated with suicide (interpersonal relationship, various stressors, substance abuse, psychiatric illness, physical illness, change of behavior before suicide, suicide note, method used, and previous attempts, etc.).
3. **Semi-structured performa for treatment details:** This performa was used to collect information regarding details of treatment, if any, sought before suicide.

### Procedure

A medical social worker and a qualified counselor (clinical psychologist) visited the families and interviewed the relatives of the deceased. The information was obtained from a “key informant” of the suicide victim, who was a spouse, offspring, sibling, parent, or any other near or dear one who was living with the deceased for at least the past 2 years. Semi-structured pro forma numbers 1 to 3 were completed by the research team. Information to elucidate the circumstances in which suicide had occurred was also recorded. Out of 130 cases, families of only 101 suicide victims could be contacted, and others could not be traced due to wrong address, shift of residence; while four families refused to participate. Data so collected was analyzed statistically using descriptive statistics.

## RESULTS

In the present study, majority (59.4%) of suicide victims were in the age group of 20 to 29 years, followed by age group 30 to 39 years (14.8%). Males (57.4%) slightly outnumbered females (42.57%) in our study. A large number of subjects (32.6%) were educated up to high school level, followed by graduates (24.7%). Majority of the subjects were unemployed (55.4%); and among the employed category, 39.6% of the suicide victims were semiskilled employees and 4.9% of the subjects were professionals. As many as 57.4% of the subjects were unmarried, and 40.5% of the subjects were married. As many as 70.2% of the victims had an urban background, while 9.89% were from rural areas and 19.8% were from suburban area. About half (50.4%) were from low-income group, 24.7% were from low-middle income group, and 21.7% of the subjects were from the middle income group.

In this study, 57.4% of the subjects had migrated from other parts of India and abroad, and 22.4% of the migrated population was from Uttar Pradesh (U.P.). This was followed by Punjab (17.2%), Himachal Pradesh (10.3%), Bihar (6.8%), and Haryana (6.8%). From abroad, the migrated subjects came from Nepal (8.6%). According to the place of migration, a large number of suicide victims were from Aligarh (18.9%), a district of U.P. This was followed by the migrants from Kathmandu (8.6%). Among the migrated sample, a large number of subjects were unmarried (53.4%) and educated up to 12^th^ class (63.7%). On chi-square analysis, there was no significant difference between the migrant population and the local population with regard to age, marital status, and educational qualifications (*P* > 0.05).

Hanging was the most common method (72.2%), while 15.8% of the victims used poisoning as a method of suicide. Self-immolation was used by 5.9% of the sample, and 2% of the subjects jumped from roofs of their houses. As many as 50.4% of the subjects committed suicide during daytime (8 A.M.-5 P.M.), 23.7% in early morning (midnight to 4 A.M.), and 13.8% between 8 P.M. and midnight.

As many as 8.9% of the victims had past history of attempting suicide. Psycho-social stressors were found in 61 (60.3%) suicide victims; while 47.5% of the subjects believed interpersonal stressors were the cause of suicide, and 8.9% of suicide victims had financial stressors. Occupational stressors were found in 3.9% of the suicide victims [Table 1]. Ten (9.9%) suicide victims had clashes with law, and 19 (18.8%) victims had pending court litigations. Psychiatric illness was found in 34 (33.6%) cases. However, out of them only 16 (48.5%) suicide victims had sought treatment prior to the attempt. Fourteen subjects were on regular treatment; out of them, only 4 subjects were taking treatment from mental-health professionals. As many as 16.8% of the subjects had epilepsy, while 11.9% of them had depressive episode. Alcohol/substance abuse was found in 23.7% of the subjects.

**Table 1:** Psychosocial factors related to suicide (N = 101)

	N	%
Mode of suicide		
Hanging	73	72.2
Poison	16	15.8
Self immolation	6	5.9
Jumped	2	1.9
Drowning	2	1.9
Stabbing	1	0.9
Shooting	1	0.95
Time of suicide		
12 A.M.-4 A.M.	24	23.7
4 A.M.-8 A.M.	8	7.9
8 A.M.-5 P.M.	51	50.4
5 P.M.-8 P.M.	4	3.9
8 P.M.-12 A.M.	14	13.8
Earlier attempts		
Yes	9	8.9
No	92	91.08
Psychosocial stressors	61	60.3
Subtypes		
Interpersonal stressors	48	47.5
Occupational stressors	4	3.9
Financial stressors	9	8.9
Psychiatric illness	34	33.6
Alcohol/substance use	24	23.7

Behavioral change before attempt of suicide was seen in 57.4% of the subjects. Among the behavioral changes, 35.6% of the subjects became withdrawn, with loss of interest in the surroundings; and 15.8% were irritable and aggressive [Table 1]. Six (5.8%) suicide victims gave hint about their intention to commit suicide to their family members and friends. Half of these cases talked to their spouses about the suicide. Suicide note was found only in five subjects.

A large number of migrant subjects (27.5%) had disturbed interpersonal relationship with family members. There were more job-related stressors in this population in comparison to the local population. Job changes were seen in 6.8% of the migrant subjects.

## DISCUSSION

There is a steep rise in the number of suicides all over the world. Currently, it is becoming a matter of concern for mental-health professionals on account of its increasing incidence. Our study reports on 101 victims who committed suicide in 2003 in the Union Territory, Chandigarh. The study population was comprehensive as we had included consecutive suicides in Chandigarh in the year 2003. A community team comprising of a medical social worker (Master in Social Work) and a qualified counselor (Master in Psychology) visited the families and interviewed a key informant of the deceased, and thus the information available was reliable. Psychological autopsy approach was used in this study to elucidate the nature and causes of completed suicide. Similar approach has been employed in earlier Indian and international studies.[2,4]

In the present study, a male preponderance was observed in the subjects who attempted or committed suicide (male; 57.4% vs female; 42.6%). The commonest age group committing suicide in our study was in the third decade (59.4%), followed by the fourth decade. Shukla *et al.*[9] and other Indian workers[8,9,11–14] have reported similar findings. Ponnudurai,[14] after reviewing 12 studies on suicidology from different parts of India, has concluded that second and third decade of life seems to be the most susceptible period for Indian suicides. In our study, unmarried victims constituted 57.4% of the total sample. A large number of subjects in our study belonged to low socioeconomic category, and a high percentage of subjects were unemployed. Chandigarh being the capital of two states and being a union territory also, a large number of people from neighboring states migrate here in search of a job. In the present study, a large number of suicide victims (57.4%) were not permanent residents of Chandigarh and had migrated from other states. They were living on the outskirts of Chandigarh in slum colonies. A recent or repeated change of residence is often found to be more prevalent among people who kill themselves than among other people. These findings suggest the importance of social ties in relation to people's propensity to commit suicide. Similar findings were reported in a number of earlier studies.[15,16] Our study endorses the previous research findings that unmarried persons, unemployed persons, and persons with poor socioeconomic status are at high risk of committing suicide.[15,17–18] A job can provide many things. It may offer not only money but also social contacts, social position, support possibilities, and stable routines. The connection between unemployment and suicide is largely dependent on what exactly unemployment means for the individual. In the current scenario in India, employment opportunities are shrinking, and various government and non-government agencies must frame and implement adjustable policies of self-employment to address this emerging problem. The burden of suicide in the northern region of India can no longer be ignored. Recent reports of large numbers of farmers committing suicide in Punjab have raised many eyebrows. It has been observed that the farmers who committed suicide were under huge debts, and the income from agriculture was not adequate to repay the borrowed money. In the absence of any help, these farmers perhaps chose to end their lives. This phenomenon is imposing a challenge not only to mental-health professionals but also to political, religious, and social reformers. Suicide can be the tragic end point of interplay of a wide array of factors, including biological, genetic, social, cultural, psychological, and behavioral factors.[3] It is imperative that multiple approaches be adopted for intervention with the goal of suicide prevention. Government of India enacted the National Rural Employment Guarantee Act on August 25, 2005. Starting April 1, 2008, this act now in 330 districts, is being extended to cover the remaining districts across India. The Government of India guarantees employment upto 100 days to every rural household that demands work. In future, proper implementation of such schemes can prove to be beneficial in tackling the growing menace of unemployment.

Hanging was the commonest method used by the subjects in this study. Similar findings were reported by many authors from Indian studies.[8,9] Some hospital-based Indian studies had revealed that insecticide poisoning was the commonly employed method in committing suicide.[19–21] The present study was conducted in a city where majority of the population is non-agriculturist. Chandigarh being the capital of two states (Punjab and Haryana), law-enforcing agencies implement strict rules and regulations with regard to sale of insecticides in this region. In this study, the time of committing suicide among majority of suicide victims was during office hours (8 A.M.-5 P.M.). The suicide victims might have been alone in their homes during this time to complete the act. In hanging, chances of successful suicide are high; as compared to other methods, no investment and preparation are required for hanging.

Interpersonal stress appears to be the common cause of suicide in this study. Similar observations have been made by several other researchers.[4,8–9] Psychiatric illness was found in 33.6% (*n* = 34) of the subjects in this study. This figure was higher than the reported rate (23%) in a previous Indian study.[9] We found that 11.9% (*n* = 12) of the victims had depressive episode, 23.7% had alcohol/substance abuse, and 16.8% of the subjects had epilepsy. As many as 57.4% of the subjects had shown behavioral change before the suicidal act. In our study, less than half (48.5%) of the subjects who were suffering from mental illness and alcohol/substance abuse sought treatment prior to the act, and only 11.8% sought treatment from mental-health professionals. In many international studies, psychiatric illness has been thought to be the most important cause of suicide. In these studies, the proportion of suicide victims with psychiatric illness ranged from 73% to 100%.[2–3] In Chandigarh, there are many public health dispensaries which cater to the general health needs of the local residents. However, general physicians working in these dispensaries have very little exposure to psychiatry during their undergraduate training. In Chandigarh, there is a need of a suicide prevention program to decrease the incidence of suicide in this region. Physicians’ and general practitioners’ education for assessment of suicide risk may be organized through professional medical associations, for example, the Indian Medical Association (IMA), Association of Physician of India (API) etc.

Our study suggests that specific focus in suicide prevention strategies should be on the migrant population. Family education programs for early identification of mental illness and risk of suicide are urgently required in India. There is a need to bridge the treatment gap in psychiatric care services. Another need is to improve the quantum of psychiatry training in the medical undergraduate curriculum. This should include suicide, its understanding and prevention.
